# In and out of the rRNA genes: characterization of *Pokey* elements in the sequenced *Daphnia* genome

**DOI:** 10.1186/1759-8753-4-20

**Published:** 2013-09-23

**Authors:** Tyler A Elliott, Deborah E Stage, Teresa J Crease, Thomas H Eickbush

**Affiliations:** 1 Department of Integrative Biology, University of Guelph, Guelph, ON N1G 2W1, Canada; 2 Department of Biology, University of Rochester, Rochester, NY 14627, USA; 3 Department of Biology, Butler County Community College, Butler, PA 16002, USA

**Keywords:** Transposons, *Daphnia*, *Pokey*, Ribosomal DNA, Insertion specificity

## Abstract

**Background:**

Only a few transposable elements are known to exhibit site-specific insertion patterns, including the well-studied R-element retrotransposons that insert into specific sites within the multigene rDNA. The only known rDNA-specific DNA transposon, *Pokey* (superfamily: *piggyBac*) is found in the freshwater microcrustacean, *Daphnia pulex*. Here, we present a genome-wide analysis of *Pokey* based on the recently completed whole genome sequencing project for *D. pulex*.

**Results:**

Phylogenetic analysis of *Pokey* elements recovered from the genome sequence revealed the presence of four lineages corresponding to two divergent autonomous families and two related lineages of non-autonomous miniature inverted repeat transposable elements (MITEs). The MITEs are also found at the same 28S rRNA gene insertion site as the *Pokey* elements, and appear to have arisen as deletion derivatives of autonomous elements. Several copies of the full-length *Pokey* elements may be capable of producing an active transposase. Surprisingly, both families of *Pokey* possess a series of 200 bp repeats upstream of the transposase that is derived from the rDNA intergenic spacer (IGS). The IGS sequences within the *Pokey* elements appear to be evolving in concert with the rDNA units. Finally, analysis of the insertion sites of *Pokey* elements outside of rDNA showed a target preference for sites similar to the specific sequence that is targeted within rDNA.

**Conclusions:**

Based on the target site preference of *Pokey* elements and the concerted evolution of a segment of the element with the rDNA unit, we propose an evolutionary path by which the ancestors of *Pokey* elements have invaded the rDNA niche. We discuss how specificity for the rDNA unit may have evolved and how this specificity has played a role in the long-term survival of these elements in the subgenus *Daphnia*.

## Background

Transposable elements (TEs) are found in nearly all organisms and often comprise substantial portions of eukaryotic genomes [1]. Many TEs insert into locations throughout the genome, while others insert preferentially into specific sequences. A site preferred by non-long terminal repeat (non-LTR) retrotransposons is the locus encoding rRNA [2]. *Pokey* is the only example of a DNA transposon known to insert specifically in rDNA. *Pokey* inserts into the same 28S gene region that is highly targeted by non-LTR elements [3]. Insertion of any of these elements is expected to disrupt the production of functional rRNA from the inserted units.

rDNA is comprised of hundreds to thousands of units arrayed in tandem encoding one copy each of the core 18S, 5.8S and 28S rRNAs. The many copies of each rRNA gene show high sequence identity, the product of recombinational processes termed concerted evolution (reviewed in [2]). The primary mechanism conferring high identity between copies is unequal crossing over, which also generates the large variation in rDNA copy number observed between members of the same species [4]. The combined processes of concerted evolution and selection against inserted units require that any element with a long-term presence in the rDNA unit regularly generate new insertions to avoid being eliminated from the locus [4,5].

*Pokey* elements are members of the *piggyBac* superfamily of DNA-mediated TEs that insert into TTAA target sequences [3,6]. This element was first identified in the cladoceran crustacean *Daphnia pulex*, and is now known to be widespread throughout the subgenus *Daphnia*. Unlike most DNA TEs, *Pokey* elements have undergone stable vertical inheritance for millions of years [7]. To the best of our knowledge, the only other organisms in which open reading frames (ORFs) similar to those in *Pokey* have been found are the silkmoth *Bombyx mori*[8], the tunicate *Ciona savignyi*[9], and the rotifer *Adineta vaga*[10]. *Pokey* elements have been found at multiple TTAA insertion sites throughout the genome and thus, like other *piggyBac* elements, appear to require little additional conservation of target sites [3,11]. Nevertheless, *Pokey* have been repeatedly found at just one location in the 28S genes despite the presence of over 30 TTAA motifs in the entire rDNA unit. While this finding might suggest that properties in addition to TTAA are preferred for *Pokey* insertion, the frequency of independent *Pokey* insertions in the rDNA locus is not known. Thus, it is unclear whether rDNA acts as a sink or source for *Pokey* elements, or whether there is free and on-going exchange between *Pokey* elements in and outside the rRNA genes.

In this study, we used the original sequencing reads available from the *Daphnia* genome sequencing project, available at the Trace Archives at GenBank, as well as the annotated scaffold sequences to study *Pokey* elements and their interactions with 28S genes. The *Pokey* elements are divided into two divergent lineages each possessing a unique inverted terminal repeat (ITR) structure. Both lineages carry repeated copies of a segment from the intergenic spacer (IGS) region of the rDNA unit. In addition, two lineages of non-autonomous miniature inverted repeat transposable elements (MITEs) are present at the *Pokey* site in 28S genes, and elsewhere in the genome. Finally, weak target sequence preferences for *Pokey* and the MITEs were found that are consistent with the site that is targeted in the 28S gene. We suggest that *Pokey* elements have evolved specificity for their 28S gene insertion site and their presence at this site has played a key role in their long term survival in *Daphnia* by acting as a source for *Pokey* and their MITEs throughout the genome.

## Results

### rDNA sequence variation

**Figure 1:**
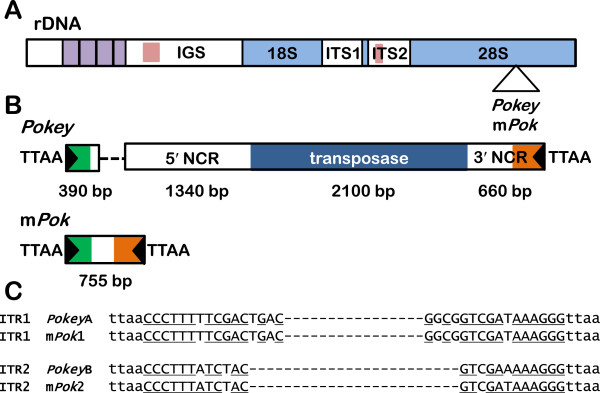
**The structure of rDNA and its transposons in the *Daphnia *genome sequence. (A)** The rDNA is an array of transcription units repeated in tandem and separated by the intergenic spacer (IGS), which contains a repeated sequence (purple boxes), as well as non-repeated regions. Pink boxes in the IGS and internal transcribed spacer (ITS)2 indicate the position of sequences found in the 5′ noncoding region (NCR) of *Pokey* elements. **(B)** The structure of *Pokey* and *Pokey*-derived miniature inverted repeat transposable elements (m*Pok*), which insert in a specific TTAA site in 28S rRNA genes. The approximate length of each region of a canonical autonomous *Pokey* element is given below the diagram. The black triangles at each end represent the inverted terminal repeats (ITRs). The dashed line in *Pokey* represents the highly length-variable region composed of both repetitive and unique sequences (more detail is provided in Figure 5). The green and orange regions correspond to regions that are similar in *Pokey* and m*Pok,* the sequences of which are shown in Figure 2. **(C)** Sequence of the two types of ITRs. Complementary regions between the imperfect 5′ and 3′ ITRs are underlined. The TTAA target site duplication is shown in lower case.

Assembly of a consensus rDNA repeating unit from the *Daphnia* genome revealed a gene organization typical of most eukaryotes (Figure 1A, Additional file 1). The IGS separating transcription units in *Daphnia* starts with an 840 bp non-repetitive region, followed by a series of 323 bp repeats, and ends in a non-repetitive 3,115 bp region. The last region should include an external transcribed spacer, but the transcription start site is not known. Ambrose and Crease [12] have shown that the 323 bp repeats are composed of two subrepeats of 200 bp and 123 bp, which usually (but not always) alternate with each other. Most (58%) IGSs in the genome sequence contain three 123-bp and four 200-bp repeats with the remaining IGSs containing more copies of each repeat.

Concerted evolution is expected to maintain very high sequence identity among all copies of the rDNA unit. The rDNA transcription units (external transcribed spacer through the 28S gene) in sequenced genomes of *Drosophila*[13] and *Nasonia*[14] contain from 3 to 18 sites in which sequence variants are present in over 3% of units. In contrast, the *Daphnia* rDNA transcription unit and a 500 bp non-repetitive region from the IGS contain no sequence variants at the 3% threshold. This especially low level of rDNA variation is consistent with the very high level of homozygosity at allozyme and microsatellite markers observed in the sequenced *Daphnia* isolate [15], the low level of sequence variation in 28S genes from *D. pulex* in natural populations [16], and the high rate of recombination observed in the rDNA of a closely related species, *Daphnia obtusa*[17].

### *Pokey* elements in 28S genes and the genome

A consensus sequence for *Pokey* copies was assembled from the original sequence reads of the *D. pulex* genome (Additional file 1). We also identified 69 elements containing intact ITRs at both ends from the annotated genome scaffolds at wFleabase. We aligned these sequences to two copies of *Pokey* elements from *D. pulicaria* rDNA, which were designated pc*Pokey*S (5 kb) and pc*Pokey*L (6.6 kb) [3]. As shown in Figure 1B, the *Pokey* elements contain either 12 or 16 bp imperfect ITRs, a 5′ non-coding region (NCR), an ORF encoding a putative transposase, and a 3′ NCR. The *D. pulex* copies were up to 9,800 bp in length (Additional file 2) with the majority of the length variation occurring in the 5′ NCR (discussed below). Excluding this repetitive region, the canonical *Pokey* element is approximately 4,500 bp.

We also identified an additional 91 incomplete sequences from 400 to 4,400 bp in length that lack either the 5′ or 3′ ITR, or both. The total number of *Pokey* elements based on these genomic searches was 160, similar to the 175 estimated by comparing the depth of coverage of *Pokey* sequence reads to the average coverage of single copy genes [15]. We estimate that six of the 175 copies are inserted into 28S genes and they all have the 12-bp ITRs.

**Figure 2:**
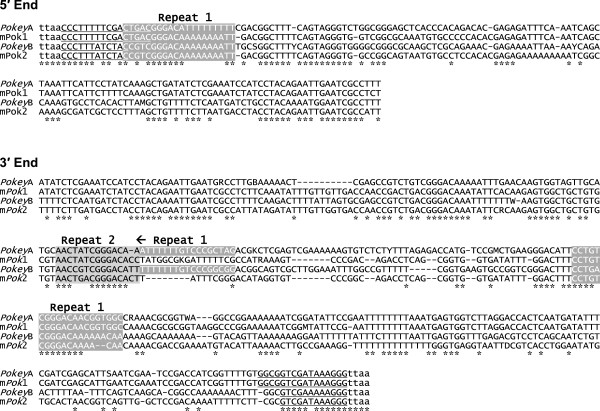
**Partial alignment of *Pokey *and m *Pok *consensus sequences.** Nucleotides conserved across all four sequences are marked with asterisks. The inverted terminal repeats are underlined. Repeat 1 and 2 refer to sequences shown in Additional file 6. B = C, G or T, M = A or C, R = A or G, W = A or T. m*Pok*, *Pokey*-derived miniature inverted repeat transposable element.

A second type of sequence was also found at the *Pokey* insertion site in 28S genes and elsewhere in the genome. These elements were approximately 750 bp in length and contained sequences corresponding to the ends of the *Pokey* elements (Figure 1B). These shorter elements could be divided into two groups that contain the same imperfect 12 or 16 bp ITRs found in full-length *Pokey* elements, and thus are designated as MITEs. Sequence identity between the *Pokey* and MITEs extends for 160 bp at their 5′ ends and 350 bp at their 3′ ends (Figure 2). These regions contain repeat sequences that have been found in other *piggyBac* elements [18]. The central 250 bp region of the MITEs has no readily observed similarity to that of the *Pokey* elements.

Like the *Pokey* elements, MITEs found outside 28S genes also target TTAA sites, suggesting that they use the transposase of *Pokey* elements. We hereafter refer to these MITEs as m*Pok*. About 25 to 30 copies of these m*Pok* were found in 28S genes, all with 12-bp ITRs. The total genome contains 90 to 110 copies with 60 m*Pok* sequences in the assembled scaffolds (Additional file 2).

### Cluster analysis of *Pokey* and m*Pok* elements

**Figure 3:**
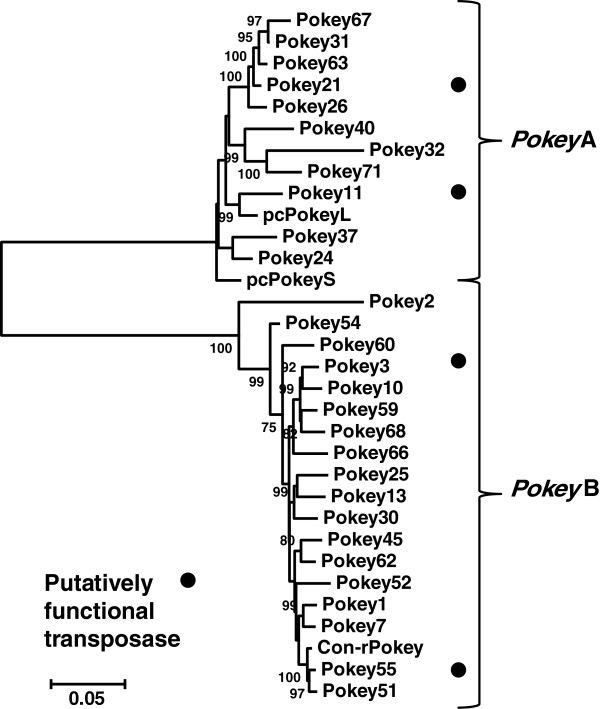
**Unrooted Neighbor-joining tree of full-length *Pokey *elements from the *Daphnia *genome.** The 27 elements containing putative transposase genes and less than 5% ambiguous bases, and the consensus rDNA element (Con-r*Pokey*) are included in the tree. The elements form two well-supported clusters that correspond to the inverted terminal repeat (ITR)1 and ITR2 sequences (Figure 1C). All positions containing gaps and missing data were eliminated in pairwise comparisons. Bootstrap values greater than 70 are indicated at the nodes of the tree.

A Neighbor-joining (NJ) tree was constructed from the consensus rDNA *Pokey* sequence, the pc*Pokey*S and L sequences from *D. pulicaria* and 29 *Pokey* elements from the assembled genome scaffolds of *D. pulex* that contained full-length transposase sequences and less than 5% ambiguous base-calls. The length-variable region of the 5′ NCR (Figure 1B) was omitted from this analysis. The tree revealed two clusters with high bootstrap support, which will be referred to as the *Pokey*A and B families (Figure 3). The *Pokey*A cluster contains the two pc*Pokey* elements described from *D. pulicaria*[3]. The *Pokey*B cluster contains a second paralogous lineage of *Pokey* elements previously identified by Penton and Crease [7] from *D. obtusa* (Additional file 3). All *Pokey*A elements contain the 16-bp ITR1, while *Pokey*B elements have the 12-bp ITR2. Average sequence divergence between the 11 *Pokey*A elements is 5.9% while average divergence between the 18 *Pokey*B elements is 5.0% (Table 1). Divergence between the two groups averages 39.9%. Based on the sequence of their ITRs, 11 (15.9%) of the 69 elements obtained from the annotated scaffolds are *Pokey*A while the remaining 58 (84.1%) are *Pokey*B (Additional file 2).

**Table 1:** **Sequence divergence between *Pokey *elements from the *Daphnia *genome**

**Lineage**	***Pokey*A**	***Pokey*B**	**m*Pok*1**	**m*Pok*2a**	**m*Pok*2b**
***Pokey*A**	0.059	0.399			
***Pokey*B**		0.050			
**m*Pok*1**			0.032		
**m*Pok*2a**			0.249	0.022	
**m*Pok*2b**			0.323	0.200	0.204

Estimates were calculated using the Kimura 2-parameter and pairwise deletion options in MEGA4. Estimates are based on separate alignments of *Pokey*A and *Pokey*B elements (28 sequences) and of m*Pok* (61 sequences). m*Pok*, *Pokey*-derived miniature inverted repeat transposable element.

**Figure 4:**
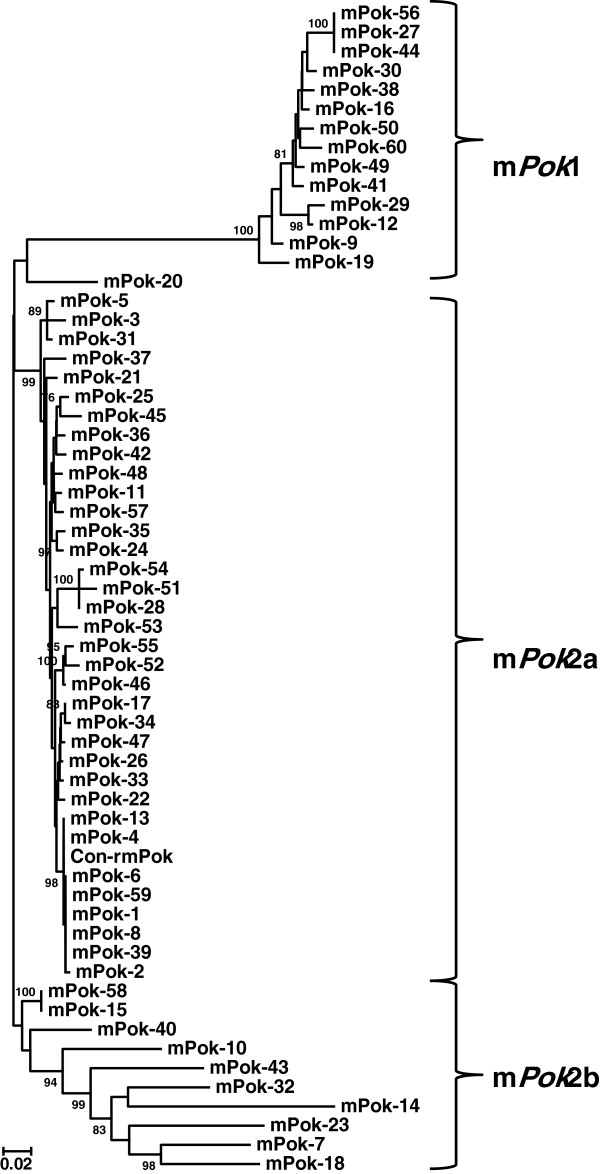
**Unrooted Neighbor-joining tree of 61 m*Pok *sequences from the *Daphnia *genome.** Con-rm*Pok* is the consensus element in rDNA. The elements form two main clusters, denoted m*Pok*1 and m*Pok*2 that correspond to elements with inverted terminal repeat (ITR)1 and ITR2 sequences, respectively (Figure 1C). All positions containing gaps and missing data were eliminated in pairwise comparisons. Bootstrap values greater than 70 are indicated at the nodes of the tree. m*Pok*, *Pokey*-derived miniature inverted repeat transposable element.

An NJ tree was also constructed with all 60 m*Pok* sequences identified in the assembled scaffolds and the consensus rDNA m*Pok* sequence. Two clusters with high bootstrap support were again observed (Figure 4), one sharing the 16 bp ITR1 with *Pokey*A (designated m*Pok*1) and the other sharing the 12 bp ITR2 with *Pokey*B (designated m*Pok*2). m*Pok*2 elements (46 copies) are over three times as numerous as m*Pok*1 elements (14 copies). Intragroup sequence divergence for m*Pok*1 is only 2.2%. In the case of m*Pok*2, there is a large cluster of elements (m*Pok*2a, Figure 4) with low average sequence divergence (3.2%) and a second group (m*Pok*2b) with much higher divergence (20.4%, Table 1). Inspection of the m*Pok*2b sequences reveals few intact ITRs and numerous insertions and deletions suggesting that they represent older copies of m*Pok*2 that are no longer able to transpose. Divergence between m*Pok*1 and m*Pok*2a is 24.9% (Table 1), somewhat lower than the divergence estimates between full length *Pokey*A and *Pokey*B elements.

### Characterization of the *Pokey* transposase

The ORF from the pc*Pokey*L element was originally reported by Penton and colleagues [3] to be 1,461 bp encoding a protein of 487 amino acids. However, this coding region was suggested to contain a 68 bp intron (Y Bigot, personal communication), which when spliced from an RNA transcript would enable the production of a 668 amino acid protein. *Pokey* elements from *D. pulex* also appear to have this intron, which ranged in size from 68 to 74 bp in *Pokey*A and from 79 to 84 bp in *Pokey*B. Analysis of *Pokey* RNA transcripts by RT-PCR confirmed that the putative intron sequence can be spliced out [19].

*Pokey*A *and Pokey*B transposase genes encode conserved motifs shared among the transposase genes of diverse *piggyBac* elements [20]. These include a DDD (aspartic acid) motif (amino acid residues 436, 544 and 659) that is considered essential for transposase activity, an imperfect zinc finger motif that is believed to be either a chromatin-interacting Plant Homeo Domain or a protein-protein interaction domain, and a putative nuclear localization signal. Keith and colleagues [20] identified a fourth D residue C-terminal to the catalytic DDD triad. When they mutated this charged D to an uncharged N (asparagine) in a *piggyBac* construct, they observed a significant reduction in the transposition rate. This fourth residue is N instead of D in the *D. pulicaria* and *D. pulex Pokey* elements (Additional file 4). Partial sequences of *Pokey* transposase genes from other species in the subgenus *Daphnia*[7] all encode an N at this site.

Of the 69 elements identified from the assembled scaffolds, two *Pokey*A and two *Pokey*B elements were identified that may encode transposition-competent transposases (identified on the NJ tree in Figure 3). The ORF of these elements lacked premature stop codons and contained all features known or inferred to be important for the transposition of *piggyBac*.

### Repeated sequences in *Pokey* and m*Pok*

**Figure 5:**
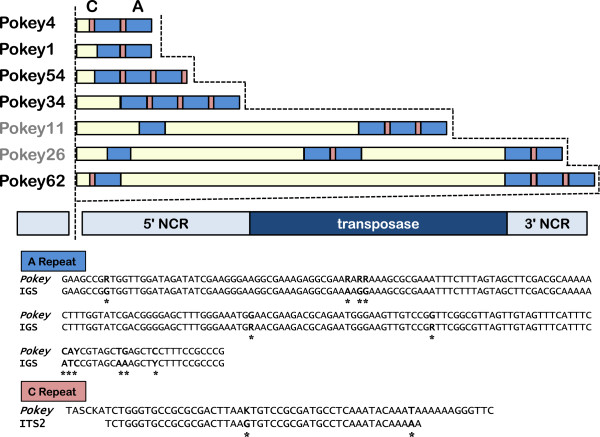
**The 5′ length-variable region of *Pokey *elements from the *Daphnia *genome.** Examples of the 5′ length-variable region containing **A** and **C** repeats are drawn to scale above the canonical element, also drawn to scale. *Pokey*A element names are in grey, and *Pokey*B element names are in black. The **A** repeats (approximately 200 bp) are similar to a sequence in the *Daphnia* intergenic spacer (IGS) while **C** repeats (approximately 50 bp) are similar to a sequence in the *Daphnia* internal transcribed spacer (ITS)2. Asterisks and bold type indicate variable positions in the aligned sequences. NCR, non-coding region. K = G or T, R = A or G, S = C or G, Y = C or T.

Penton and colleagues [3] noted the presence of a 200-bp repeat sequence (A repeats) in the 5′ NCR of *D. pulicaria Pokey* elements that was derived from the IGS region of the rDNA unit. We also observed A repeats in the *Pokeys* from *D. pulex* and note that they are usually preceded by a 48 bp sequence derived from ITS2 (Figure 5; see Figure 1 for the location of these sequences in the rDNA unit). The ITS2 repeat was termed C to differentiate it from an IGS-derived sequence previously designated as B in the pc*Pokey*L element [3]. All but one *Pokey* element from the annotated scaffolds contain both A and C repeats with their copy number varying between 2 and 5 per element. Due to the possibility of assembly errors in repeat regions, we cannot be certain of the exact repeat configuration of each element. However, the evidence does suggest these regions are highly variable among elements. In addition, large tracts of additional sequences derived from areas of the *Daphnia* genome outside the rDNA units were inserted between the A repeats of several *Pokey* elements (Figure 5) suggesting that the 5′ NCR frequently acquires non-element sequences from the genome.

**Figure 6:**
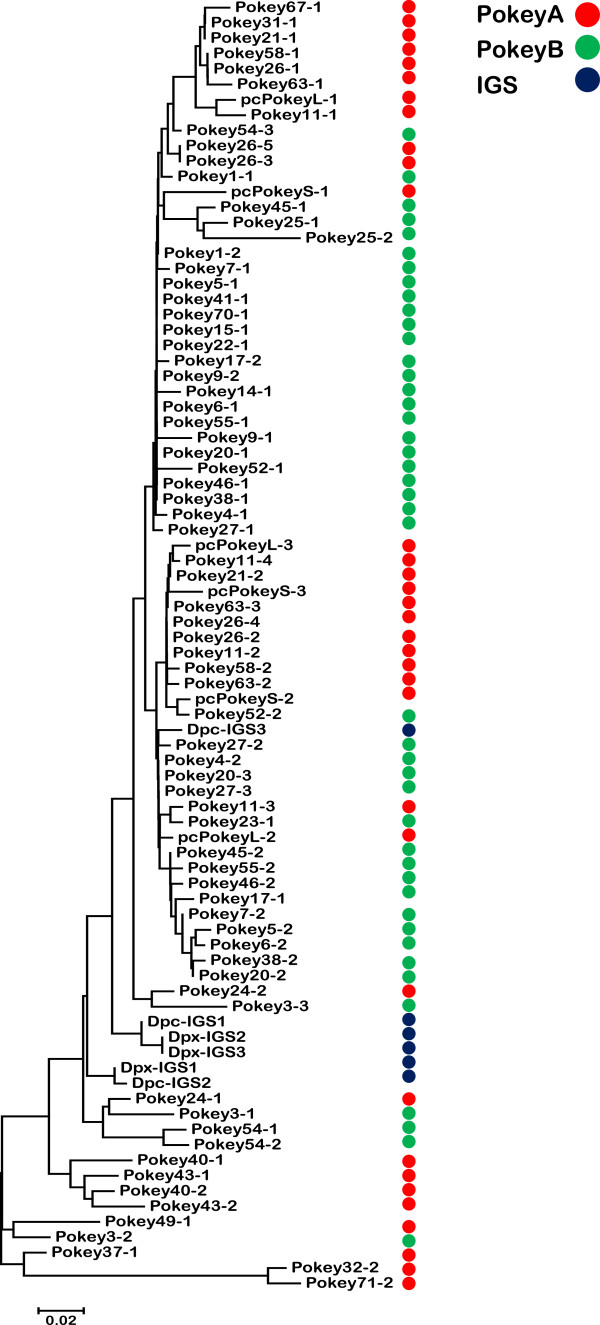
**Unrooted Neighbor-joining tree of A repeats from the intergenic spacer (IGS) and *Pokey *elements in *D. pulex *and *D. pulicaria*.** The tree was generated from the alignment in Additional file 5. All positions containing alignment gaps and missing data were eliminated in pairwise sequence comparisons.

We aligned the A repeat region of three *D. pulex* and three *D. pulicaria* ribosomal IGS sequences [12] to A repeats from all available *Pokey* elements (Additional file 5) and generated an NJ tree (Figure 6). The IGS sequences do not cluster separately from the *Pokey* repeats, nor do repeats from *Pokey*A and *Pokey*B elements form separate clusters relative to one another. Mean sequence divergence among the A repeats from all *Pokey* elements is only 5.3% (range 0 to 23.9%). In comparison, intraspecific sequence divergence in the region of the *D. pulex* and *D. pulicaria* IGS similar to A repeats is 1.8% [12]. This high sequence identity among the A repeats of the *Pokey* elements is in sharp contrast to the transposase sequences where mean nucleotide sequence divergence between the *Pokey*A and B families is nearly 40%. These findings suggest there have been repeated exchanges between the *Pokey*A and *Pokey*B elements and the IGS sequences of the rDNA units.

In addition to A and C repeats, there are three other short repeated sequences in the 5′ and 3′ NCR of *Pokey* elements from the *Daphnia* genome that are shared with the m*Pok* elements (Figure 2 and Additional file 6). Some of these repeats may correspond to repeat sequences previously found in other *piggyBac* elements, such as the one diagrammed in Additional file 6 from *Trichoplusia ni*[18].

### Target site preferences for *Pokey* and m*Pok*

Previous characterization of *Pokey* target sites found no preference aside from the requisite TTAA observed for all *piggyBac* elements [21-23]. However, in contrast to *piggyBac* elements, about 10% of the *Pokey* and m*Pok* insertions, all oriented in the 5′ to 3′ direction, were found with target site duplications other than TTAA (Table 2). These other insertion sites were either TTAT or ATAA suggesting the only essential nucleotides are the middle T and A. Insertion of *piggyBac* elements into non-TTAA sites has also been observed in transposition assays in bat (7.2%, [24]) and human cell lines (2.4%, [25]). In both cases, the alternate sites contained the middle T and A.

**Table 2:** **Target site duplications flanking *Pokey *elements in the *Daphnia *genome sequence**

**Target site duplications**	**% of insertions**
TTAA	88.13
TTAT	5.93
ATAA	5.09
CTAA	0.85

**Figure 7:**
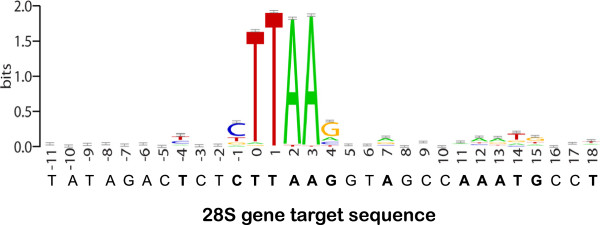
**Sequences flanking the TTAA target site of *Pokey *and *Pokey*-derived miniature inverted repeat transposable elements (m*Pok)*.** WebLogo was used to analyze 26 bp upstream and 26 bp downstream of the TTAA site. The analysis is based on 130 unique sequences upstream and 162 sequences downstream of the target site from the *Daphnia* genome. The corresponding sequence in the *Daphnia* 28S gene is shown below the graph. Positions in the 28S gene that match preferred positions in genomic insertion sites are in bold type.

Unlike insertions outside the rDNA locus, all *Pokey* and m*Pok* elements but one insert at a single site in 28S genes. The exception was an m*Pok* sequence inserted into ITS2 near the sequence that gave rise to repeat C in *Pokey* elements. The specificity of *Pokey* elements for the 28S gene site, despite the presence of over 30 TTAA sites in the rDNA unit, suggests that a larger recognition sequence could be involved in *Pokey* insertions. We therefore re-evaluated the flanking sequences of *Pokey* and m*Pok* insertions outside of 28S genes. About 23% of m*Pok* and 7% of *Pokey* copies are inserted into the TTAA flanking another *Pokey* or m*Pok* insertion (that is, they are organized as tandem repeats), and were excluded from the analysis. Visualization of preferred bases at specific sites revealed a weak preference for several bases immediately surrounding *Pokey* insertions (Figure 7). Significant sequence preferences included a C one base and a T four bases upstream (5′) of the TTAA, and a total of eight preferred bases downstream (3′) of the TTAA: a G at position 4, an A at position 7, the sequence AAATG at positions 11 to 15 and a T at position 18. Remarkably, each of these preferred bases match the *Pokey* target site in the 28S gene.

## Discussion

### *Pokey* diversity in the *Daphnia* genome

Analysis of over 160 *Pokey* and *Pokey*-like sequences from the *D. pulex* genome revealed four well-supported clusters. Two clusters of larger elements with an average size of 5,100 bp were designated *Pokey*A and *Pokey*B. The clusters have diverged in sequence by about 40%, have different ITR structures and include members that possess an intact transposase ORF. The two other clusters are MITEs, designated m*Pok*1 and m*Pok*2, because each m*Pok* element contains an ITR and other non-coding sequences corresponding to one of the full-length *Pokey* elements. Annotated *Pokey*B and m*Pok*2 elements outnumber *Pokey*A and m*Pok*1 elements by over 4:1.

Available evidence suggests that both *Pokey*A and *Pokey*B occur in *D. obtusa*[7] and thus the two lineages have likely persisted across multiple speciation events. Vertical diversification of TEs within the same genome can be driven by drift, selection, or more likely a combination of the two. Two models have been proposed. Lampe and colleagues [26] observed a loss of interaction between the ITRs and transposases of Tc1/*mariner* elements from different subfamilies with sequence divergence greater than 16%. They postulated that silencing mechanisms based on sequence similarity might create intragenomic selection that favors divergence of the transposase and ITR sequences of related TEs to escape silencing. A second possibility is that the presence of numerous non-autonomous elements drives the divergence of transposase and ITR sequences because the non-autonomous copies titrate the transposase from autonomous copies and decrease their fitness [27]. In that case, intragenomic selection might favor divergent elements whose transposases can only recognize their own ITRs.

The ability of *Pokey*A and *Pokey*B elements to cross-mobilize could be investigated using yeast excision, yeast one-hybrid and/or electrophoretic mobility shift assays to determine the strength of interaction between the transposases and ITRs of each group. Although the differences in sequence between the two ITR structures appear minor (Figure 1), Casteret and colleagues [28] demonstrated that a small number of single nucleotide changes to the ITR of the drosophilid DNA transposon *Mos1* produced significant changes in transposition rate.

The m*Pok* elements appear to be of an atypically large size (approximately 750 bp) compared to other MITEs, which can be as small as around 130 bp [29]. However, MITEs that are even larger than m*Pok* have now been discovered in phylogenetically diverse eukaryotes (reviewed in [30]) suggesting that large MITEs are more common than once thought. One mechanism to explain the origin of large MITEs is progressive internal deletion of autonomous DNA TEs and subsequent selection for increasing transposition rate among the resultant elements over time [31]. Thus, the larger size of m*Pok* elements could be a consequence of their recent evolution. While this could be true for the *mPok1* elements, which show little sequence diversity, the occurrence of highly divergent m*Pok*2b copies is not consistent with a recent origin (Figure 4). Indeed, Deprá and colleagues [32] suggested that the *Mar* MITEs in *Drosophila willistoni*, which are similar in size to the m*Pok* elements, may have originated prior to the diversification of the *willistoni* subgroup 5.7 MYA, suggesting that large size does not necessarily indicate recent origin.

### Repeated sequences in *Pokey*

An unusual aspect of the *Pokey*A and B lineages in *Daphnia* is the presence of sequences derived from NCRs of the rDNA unit. This includes an approximately 200 bp sequence from a non-repetitive region of the IGS (A repeats) and an approximately 50 bp sequence from ITS2 (C repeats) (Figures 1 and 5). *Pokey* elements contain from 2 to 5 copies of these rDNA sequences within their 5′ NCR (Figure 5). The highly recombinogenic nature of these repeats within the *Pokey* elements was first suggested by their differential spacing in pc*Pokey*S and pc*Pokey*L [3] and is strongly supported by this analysis in which particular combinations of A and C repeats are unique to only one or a few *Pokey* elements.

The acquisition of DNA to the 5′ NCR of *Pokey* does not appear to be limited to rDNA. For example, *Pokey*62 contains a unique, approximately 3,600 bp sequence of which approximately 1,100 bp is derived from sequence on a non-rDNA scaffold in the *Daphnia* genome. Thus, *Pokey* elements often acquire sequences from their host’s genome. Langer and colleagues [33] proposed that *Ds* elements could acquire host sequence if the transposase slides after binding but before cutting, or if cryptic ITR-like sequences exist downstream of an element. However, the acquisition of sequences well within the 5′ NCR of the *Pokey* elements argues against such a simple explanation (Figure 5).

What is the significance of the A and C repeats? It is possible that they have no function and that their origin was chance recombination events that had no fitness impact on *Pokey.* However, the finding that all but one copy of *Pokey* from both lineages contain these repeats suggests that they do play some role in *Pokey* activity. Possible functions of these sequences include transcription enhancers, transcription terminators to prevent the formation of aberrant rRNA read-through transcripts, or binding sequences to recruit epigenetic modifiers [34-36]. We suggest a transcription role for these repeats to be most likely as m*Pok* elements, which do not need to be transcribed to be mobilized by a *Pokey* transposase, do not have the rDNA repeats.

The most remarkable property of the A repeats is that the same sequence was retained in both the *Pokey*A and B lineages. Not only do the A repeats correspond to the highest level of sequence conservation between the two lineages, but the A repeats within the two *Pokey* lineages are as well conserved as IGS sequences undergoing concerted evolution within the rDNA unit (Figure 6). This high level of sequence identity suggests that recombination between the *Pokey* repeats and the rDNA repeats occurs on a regular basis, thus strengthening the argument that *Pokey* elements have become highly specialized for their insertion into the rDNA locus.

### Target site selection and the rDNA niche

While it is not possible to assemble the sequences of individual *Pokey* elements inserted in rDNA, it should be noted that the consensus *Pokey* sequence from rDNA is similar to an assembled non-rDNA copy that could putatively encode a functional transposase (Figure 3). Given the rapid turnover of rDNA units, *Pokey* elements within the locus should be among the newest insertions, while those outside of rDNA are a combination of new and old insertions.

*Pokey* is the only DNA-mediated TE that is known to evolve insertion specificity for the rDNA unit. Remarkably, the *Pokey* insertion site is in the same region of the 28S gene that is also the target site for a number of non-LTR retrotransposons (reviewed in [2]). Two of these elements, R2 and R5, which insert within a few base pairs of the *Pokey* site, encode related endonucleases that have an active site similar to class IIS restriction enzymes [37,38]. The R2 endonuclease has been shown to have exceptional specificity for the 30 to 40 nucleotides surrounding its insertion site [39,40]. Two other non-LTR retrotransposons, R1 and R4, insert 75 and 28 bp, respectively, downstream of the *Pokey* site. These elements encode an endonuclease with similarity to the apurinic endonuclease involved in DNA repair [41]. The endonuclease encoded by R1 has also been shown to have sequence specificity for the insertion site [41,42]. In all four cases, most copies of the element are inserted in rDNA with most copies outside rDNA inserted into sites with sequence similarity to the 28S gene target site [13,43].

The transposase of *Pokey* elements represents a third protein that has evolved specificity for this region of the 28S gene. Some of the best-studied examples of integrases that have evolved insertion specificity involve the LTR retrotransposons of yeast [44-47]. In these cases, the integrases have evolved protein-protein specificity for association with specific transcription factors or chromatin structural components rather than actual DNA sequence specificity. Such protein-chromatin interactions could also be involved in the insertion specificity of *Pokey* elements, but we are not aware of any specific chromatin components that are bound to the central region of 28S genes. Alternatively, the A repeat associated with *Pokey* elements may contain a recognition site for a nucleolar protein that helps guide into the nucleolus *Pokey* elements that have been excised and are ready for insertion.

It seems a remarkable coincidence that three different lineages of TEs have evolved specificity for the same small region of the rDNA unit. The 28S target region is highly conserved, but there are many regions of the 18S and 28S genes that are conserved across eukaryotes. We suggest either the DNA in this region is highly exposed and thus accessible to the TE machinery, a yet unknown chromatin component can be utilized by the TE in its evolution of specificity, or this is one of only a few areas of the rDNA where a TE can insert without being quickly eliminated by recombination or selected against by the synthesis of disrupted rRNA.

Based on the concordance between phylogenies of rDNA *Pokey* elements and their hosts, Penton and Crease [7] concluded that *Pokey* has undergone stable, vertical inheritance in the rDNA of species in the subgenus *Daphnia* since its origin. Thus, unlike most Class II TEs, *Pokey* elements appear to have evaded complete silencing by the host for millions of years. The unique breeding system of *Daphnia*, involving extended periods of apomictic reproduction, and the complete loss of sexuality in some lineages may have created strong selection pressure on ancestral *Pokey* elements to avoid causing deleterious mutations in their host, while still maintaining a transposition rate high enough to survive. The theory describing the interaction between TEs and asexual or partially asexual hosts predicts three possible outcomes: (1) active elements are lost, (2) the host goes extinct due to TE-induced mutation, or (3) the elements become domesticated and the threat is neutralized [48]. However, *Pokey*’s invasion of rDNA suggests a fourth outcome, the long-term persistence of active elements.

Zhou and colleagues [5] have argued that rDNA is an ideal TE niche, because it is difficult for the host to completely silence elements that have inserted into genes that must be expressed. In addition, TEs inserted in the locus are continually removed by recombination events so old copies that could interfere with the elements are eliminated. Finally, each insertion has a predictable, small effect on the fitness of the host. This effect is small because all organisms contain more than enough rDNA for the production of rRNA, and those rDNA units with insertions are usually not transcribed [49]. R2 and R1 elements, which are abundant in the rDNA of arthropods including crustaceans, have not been found in *Daphnia*. Perhaps *Pokey* elements are even better adapted for this niche in that they can be lost from the rDNA locus, but copies located outside the rDNA can on occasion be active and re-establish insertions in the locus. Indeed, individual *D. pulex* that lack *Pokey*A in rDNA have been observed, but no individuals have been observed that completely lack *Pokey* elements [11,19,50-52].

## Conclusions

In spite of what would appear to be a seemingly inhospitable location for a DNA transposon, *Pokey* has evolved specificity for a site in the 28S genes of *Daphnia*. Analysis of both the annotated *D. pulex* genome and the raw trace files revealed that rDNA units display extremely low levels of sequence variation consistent with the high rates of recombination previously observed for this locus. Indeed, *Pokey* has diversified into two lineages of autonomous elements, *Pokey*A and *Pokey*B, which appear to have persisted across multiple speciation events. While members of the B lineage are located in the rDNA of the population in Oregon that was selected for genomic sequencing [15], members of the A lineage are in the rDNA of *D. pulicaria* and *D. pulex* populations outside Oregon [3,7,52]. Both *Pokey* lineages have given rise to two parallel lineages of MITES, *mPok*1 and *mPok*2, which appear to be deletion derivatives of the full-length elements.

Part of the specificity of *Pokey* elements can be attributed to the sequence specify of the transposase itself, as the target site of non-rDNA copies bears weak sequence similarity to the 28S rRNA insertion site. However, both *Pokey* lineages possess repeat sequences derived from rDNA that vary in arrangement and copy number. These repeats may play a role in the expression of *Pokey* elements from the rDNA locus, and/or a role in insertion specificity. Whatever their function, the *Pokey* repeats are evolving in concert with each other and with the rDNA unit itself suggesting ongoing sequence exchange. It remains unknown whether *Pokey* elements in or out of the rDNA locus are most active, and what fraction of new insertions occur in rDNA. While more insertions are found outside rDNA, this could simply reflect the fact that non-rDNA insertions are more stable over time. Overall, our results suggest a complex interaction between *Pokey* and its host, and highlight the need to concentrate not only on host traits but also on traits of individual families when trying to understand the current dynamics and past evolutionary history of TEs.

## Methods

### Search for and assembly of rDNA and *Pokey* elements

The original sequencing reads of the genome sequencing project from the cladoceran crustacean *Daphnia pulex* were accessed by basic local alignment search tool (BLAST) [53,54] in the Trace Archives at GenBank [55]. In addition, BLAST searches were conducted of the assembled scaffolds at wFleaBase [56].

The search for *Pokey* elements in 28S genes was conducted in the same manner as searches for other 28S-specific TEs in rDNA [57]. Briefly, a BLAST search was conducted using the downstream region flanking the *Pokey* insertion site as the query. Reads identified in this search were examined upstream of the query region for sequences that were not 28S and thus putative TEs. Once the consensus of the TE end was acquired, iterative BLAST searches were conducted using the end of each newly acquired TE extension until the 5′ junction of the element with the 28S gene was reached. In order to identify copies present outside 28S genes, the ends of the TE consensus sequences were used as BLAST queries and the flanking sequences examined. Sequences of the putative transposase gene were analyzed using the PSORTII server [58] to identify features of the amino acid sequence.

### Cluster analysis of *Pokey* elements

*Pokey* elements were aligned using a combination of the CLUSTAL, MUSCLE and MAFFT multiple sequence alignment programs available from the EMBL-EBI website [59]. Alignments were manually adjusted in the program BioEdit [60]. Only sequences with less than 5% ambiguous bases across the aligned region and containing an ITR at both ends were used in cluster analyses. Measurements of pairwise sequence divergence were calculated using the Kimura 2-parameter method [61] in MEGA4 [62]. NJ trees [63] were also constructed in MEGA4. Bootstrap analysis was performed on 1000 pseudo-replicates for each tree [64]. The alignment of full-length elements excluded the variable repeat region between the 5′ ITR and the transposase gene. In addition, a dataset including the last approximately 1,600 bp of the 3′ end of rDNA *Pokey* elements from species in the subgenus *Daphnia*[7] was aligned with the *Pokey* elements from the *Daphnia* genome sequence and used to generate an NJ tree.

### Sequence variation in rDNA and *Pokey* elements

Sequence variation present in the rDNA transcription units and in a 500 bp region of the IGS was evaluated in the same manner as described by Stage and Eickbush [13]. Briefly, 525 bp overlapping regions of each consensus were used as BLAST queries in the trace archives. Approximately 250 reads were collected from each BLAST search and evaluated for sequence changes present in at least eight sequence reads. In order to screen out sequencing errors, sites containing sequence differences were further evaluated using the trace quality scores available through the trace archives at GenBank [55].

A total of 26 base pairs on each side of the *Pokey* insertion site of both *Pokey* and m*Pok* elements, all oriented in the 5′ to 3′ direction, were compared to determine if a preferred base is present at each position. A graphical representation of sequence conservation was made using WebLogo [65]. Only the 4 bp upstream and 15 bp downstream of the insertion contain preferred bases.

### Analysis of repeat sequences in *Pokey*

Identification of repeat sequences within *Pokey*, and comparisons between *Pokey* and rDNA were performed using Pustell DNA matrix in MacVector 10.0 (MacVector Inc., Cary, NC, USA). Default parameters were used with 80% sequence identity in a 16 bp window.

## Abbreviations

BLAST: Basic local alignment search tool; Bp: Base pair; D: Aspartate; IGS: Intergenic spacer; ITR: Inverted terminal repeat; LTR: Long terminal repeat; MITE: Miniature inverted repeat transposable element; mPok: *Pokey*-derived miniature inverted repeat transposable element; N: Asparagine; NCR: Non-coding region; NJ: Neighbor-joining; ORF: Open reading frame; rDNA: ribosomal DNA; rRNA: ribosomal RNA; RT-PCR: Reverse transcriptase polymerase chain reaction; TE: Transposable elements.

## Competing interests

The authors declare that they have no competing interests.

## Authors’ contributions

THE and TJC conceived the project. TAE analyzed *Pokey* elements from the annotated genome sequence. DES analyzed rDNA and *Pokey* elements from the trace files. All authors wrote the manuscript and approved the final version.

## Supplementary Material

Additional file 1 **Consensus sequences of the rDNA unit, *Pokey *and m*Pok* from the *Daphnia *genome sequence.** The sequences are provided in Fasta format. The highly length-variable region at the 5′ end of *Pokey* elements has been omitted and is indicated by several Xs. Click here for file

Additional file 2 **List of Pokey elements extracted from the annotated scaffolds of the *Daphnia *genome sequence.** The scaffold number (S), first nucleotide position (nt), length in bp (length) and lineage (*Pokey*A or B, m*Pok*1 or 2) is provided for each sequence. NJ, Neighbor-joining tree. Click here for file

Additional file 3 **Unrooted Neighbor-joining tree of 1600 bp sequences from the 3′ end of *Pokey *elements.** Elements from the *Daphnia* genome sequence and cloned from the rDNA of other species in the subgenus *Daphnia*[7] are included. The latter are preceded by PC. All positions containing alignment gaps and missing data were eliminated in pairwise sequence comparisons. Bootstrap values greater than 70 are shown at the nodes in the tree. Click here for file

Additional file 4 **Partial alignment of transposase amino acid sequences from *Pokey *and *piggyBac*-superfamily elements.** The three conserved catalytic aspartic acid (D) residues, the four cysteine (C) residues thought to compose the zinc-finger/Plant Homeo Domain (PHD) motif and the putative nuclear localization signal (NLS) are highlighted. The asparagine (N) residue conserved in *Pokey* transposases is highlighted in grey. Other *piggyBac* elements have D at this position. pB-Bmor, putative *Bombyx mori piggyBac* transposase; pB-Harm, *piggyBac* transposase from *Helicoverpa armigera*; pB-Xtro, *piggyBac* transposase from *Xenopus tropicalis*; pB-like-Hsap, *piggyBac* transposase-derived protein from *Homo sapiens*. Click here for file

Additional file 5 **Alignment of A repeats from the IGS and *Pokey *elements in *Daphnia pulex* and *Daphnia pulicaria.*** The sequences of 76 *Pokey* A repeats and the corresponding sequence from three ribosomal IGS from each of *D. pulex* and *D. pulicaria*[12] are provided in Fasta format. The order of the repeat within an element is given after the element name (for example, *Pokey*11-3 is copy 3 in element 11). The number of A repeats ranges from 2 to 5 per element. Click here for file

Additional file 6 **Repeated sequences in *Pokey *with similarity to *piggyBac *elements.** The approximate location of repeat sequences in *piggyBac* that lack primary sequence identity with those in *Pokey*, but occur in similar locations, are indicated for both elements. The dashed line in *Pokey* presents the repetitive region described in Figure 5. The repetitive region, 5′ NCR and transposase genes are not drawn to scale. NCR, non-coding region; tpase, transposase gene. Click here for file
