# Supplementary material for: In and out of the rRNA genes: characterization of Pokey elements in the sequenced Daphnia genome
Source: Mob DNA. 2013 Sep 23;4:20. doi: 10.1186/1759-8753-4-20 (PMC3849761; doi:10.1186/1759-8753-4-20)

Additional file 3. Unrooted Neighbor-joining tree of 1600 bp sequences from the 3′ end of *Pokey* elements. Elements from the *Daphnia* genome sequence and cloned from other species in the subgenus *Daphnia* [7] are included. The latter are preceded by PC. All positions containing alignment gaps and missing data were eliminated in pairwise sequence comparisons. Bootstrap values greater than 70 are shown at the nodes in the tree.

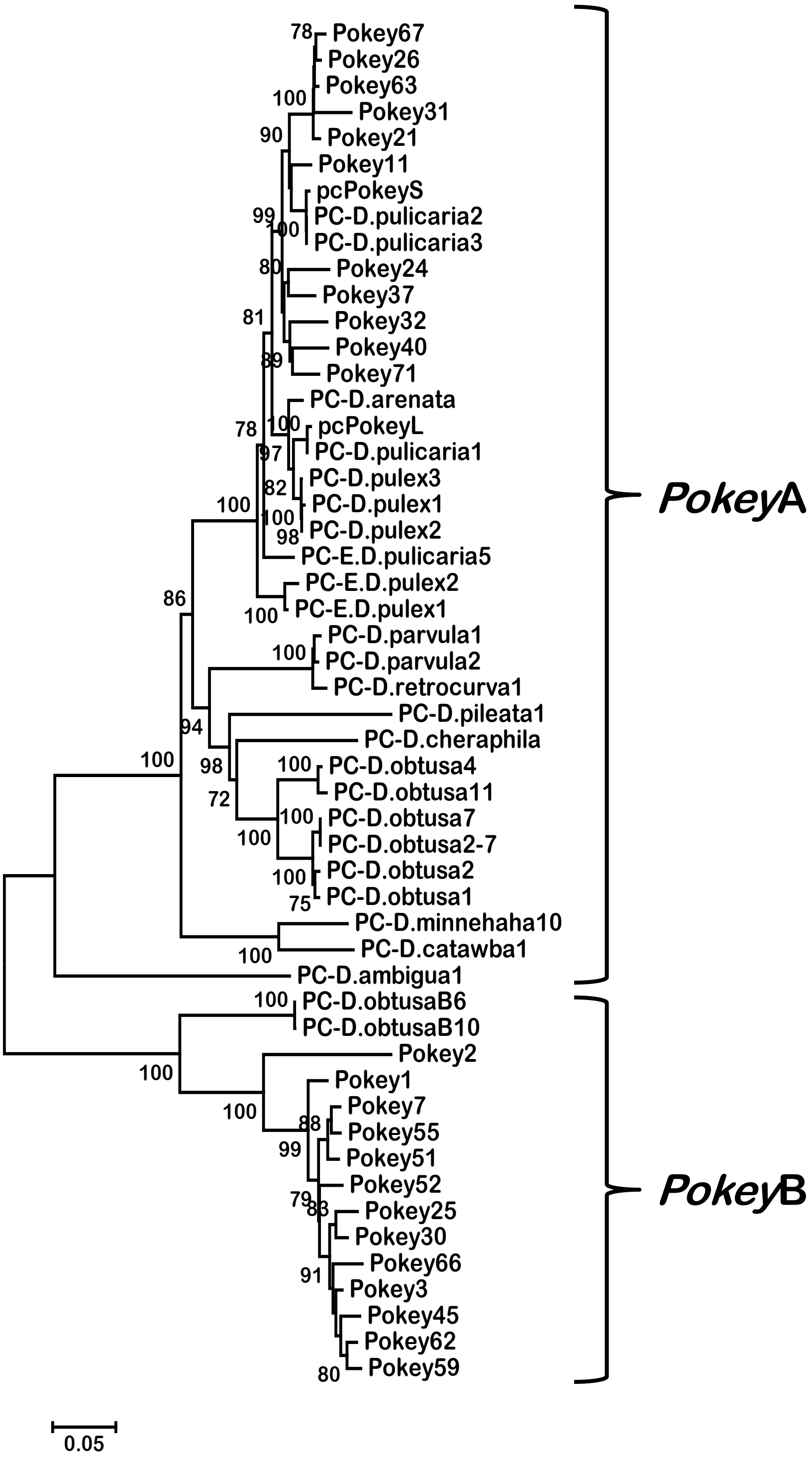

Supplement: Additional file 3 — Unrooted Neighbor-joining tree of 1600 bp sequences from the 3′ end of Pokey elements. Elements from the Daphnia genome sequence and cloned from the rDNA of other species in the subgenus Daphnia[7] are included. The latter are preceded by PC. All positions containing alignment gaps and missing data were eliminated in pairwise sequence comparisons. Bootstrap values greater than 70 are shown at the nodes in the tree. [file 1759-8753-4-20-S3.pdf]
