# Supplementary material for: In and out of the rRNA genes: characterization of Pokey elements in the sequenced Daphnia genome
Source: Mob DNA. 2013 Sep 23;4:20. doi: 10.1186/1759-8753-4-20 (PMC3849761; doi:10.1186/1759-8753-4-20)
Supplement: Additional file 4 — Partial alignment of transposase amino acid sequences from Pokey and piggyBac-superfamily elements. The three conserved catalytic aspartic acid (D) residues, the four cysteine (C) residues thought to compose the zinc-finger/Plant Homeo Domain (PHD) motif and the putative nuclear localization signal (NLS) are highlighted. The asparagine (N) residue conserved in Pokey transposases is highlighted in grey. Other piggyBac elements have D at this position. pB-Bmor, putative Bombyx mori piggyBac transposase; pB-Harm, piggyBac transposase from Helicoverpa armigera; pB-Xtro, piggyBac transposase from Xenopus tropicalis; pB-like-Hsap, piggyBac transposase-derived protein from Homo sapiens. [file 1759-8753-4-20-S4.pdf]

Additional file 4. Partial alignment of transposase amino acid sequences from *Pokey* and *piggyBac*-superfamily elements. The three conserved catalytic aspartic acid (D) residues, the four cysteine (C) residues thought to compose the zinc-finger/PHD motif and the putative nuclear localization signal (NLS) are highlighted. The asparagine (N) residue conserved in *Pokey* transposases is highlighted in grey. Other *piggyBac* elements have D at his position.

pB-Bmor = putative *Bombyx mori piggyBac* transposase
pB-Harm = *piggyBac* transposase from *Helicoverpa armigera*
pB-Xtro = *piggyBac* transposase from *Xenopus tropicalis*
pB-like-Hsap = *piggyBac* transposase-derived protein from *Homo sapiens*.

```
pcPokeyS      IRPLVKRLNERYHACRKPPRGQSIDESMVKFKGRSVLRQTMKNKPIKSGFKIWSRC-CHRGYTYKFEIYQGAR-FGEKQGRSRNNEAVERVVVDLCQPLTDQGF
pcPokeyL      IRPLVKRLNESYHVCRKPPRGQSIDESMVKYKGRSMLRQTMKNTPIKSGFKIWSRC-CLRGYTYKFEIYQGAR-FGEKQKRSRNNEAVERVVVDLCQPLTDQGF
Pokey55       IRSLVDVLNKQFNECRRPPRWQSIDESMVKFKGRSMLRKTMKGKPIKSGFKIWSRC-CSRGYTYKFEIYHGTR-IGETPKDS-NFTMVEGVVLDLCEPLAKIGH
Pokey3        IRSLVDVLNKQFSECRRPPRWQSIDESMVKFKGRSMLRQTMKGKPIKSGFKIWSQC-CSRGYTYKFEIYHGTR-IGETPKDS-NFTMVEGVVLDLCEPLAKIGH
Pokey21       IRPLVKRLNERYHACRKPPSGQSIDESMVKFKGRSVLRQTMKNKPIKSGFKIWSRC-CNRGYTYKFEIYQGAR-FGEKQGRSRNNEAVERVVVDLCQPLTDQGF
Pokey11       IRPLVKRLNERYHECRKPLRGQSIDESMVKYKGRSMLRQTIKNKPIKSGFKIWSRC-CHRGYTYKFEIYQGAR-FGEKQKRSRNNEAVERVVVDLCQPLTDQGF
pB-Bmor       FRSIFDQFVQCCQNAYSPSEFLTIDEMLLSFRGRCLFRVYIPNKPAKYGIKILALVDAKNFDVVNLEVYAGKQPSGPYAVSNRPFEVVERL----IQPVARSHR
pB-Harm       VRKIWEIFINQCRQNHVPGSNLTVDEQLLGFRGRCPFRMYIPNKPDKYGIKFPMMCAAATKYMIDAIPYLGKS-TKTN-----GLPLGEFYVKDLTKTVHGTNR
pB-Xtro       LRPLIDSLSERFAAVYTPCQNICIDESLLLFKGRLQFRQYIPSKRARYGIKFYKLCESSSGYTSYFLIYEGKDSKLDPPGCPPDLTVSGKIVWELISPLLGQGF
pB-like-Hsap  IKPVFDFLVNKFSTVYTPNRNIAVDESLMLFKGPLAMKQYLPTKRVRFGLKLYVLCESQSGYVWNALVHTGP---GMNLKDSADGLKSSRIVLTLVNDLLGQGY

pcPokeyS      VVAFDRFFTSIALLDKLREN--GVNAVGTILPSRVNQPIMTKNESNLRPDEFAAKFGGEPGTCRKGIFVW--RDTKAF--ASNYHGSNLVKVRRKQRDGSFKTK
pcPokeyL      VVAFDRFFTSIALLDKLREN--GVNAVGTILPSRVNQPIMTKNESNLRPDEFAAKFGGEPGTCRKGIFVW--RDTKAFRVASNYHGSDIVKVRRRQRDGSFRSK
Pokey55       VVAFDRFFTSIALLDELDKR--GINAVGTILKTRVGQPIFTVNESNLPQDEFVAKFGGEPGTGRKGVFIW--KDTKPFRVASNFNGSEVVKVQRKQRDGSFRTK
Pokey3        VVAFDRFFTSIALLDELDKR--GINVVGTILKTRVGQPIFTVNESNLPQDEFVAKFGGEPGTGRKGLFIW--KDTKPFRVASNFHGSEVVKVQKKQRDGSFRTK
Pokey21       VVAFDRFFTSIALLDKLREN--GVNAVGTILPSRVNQPIMTKNESNLRPDEFAAKFGGEPGTCRKGIFVW--RDTKAFRVASNYHGSDIVKVRRKQRDGSFKTK
Pokey11       VVAFDRFFTSIALLDKLREN--GVNAVGTILPSRVNQPIMTKNESNLRPDEFAAKFGGEPGTCRKGIFVW--RDTKAFRVASNYHGSDIVKVRRKQRDGSFKTK
pB-Bmor       NVTFDNWFTGYELMLHLLNE-YRLTSVGTVRKNKRQIPESFIR-TDRQPNSSVFGFQKDITLVS-----YAPKKNKVVVVMSTMHHDNSIDESTGEKQK-----
pB-Harm       NITCDNWFTSIPLAKNMLQAPYNLTIVGTIRSNKREMPEEIKNSRSRPVGSSMFCFDGPLTLVS-----YKPKPSKMVFLLSSCDENAVINESNGK--------
pB-Xtro       HLYVDNFYSSIPLFTALYCL--DTPACGTINRNRKGLPRALLDK-KLNRGETYALRKNELLAIK-----F--FDKKNVFMLTSIHDESVIREQRVGRPPKNK--
pB-like-Hsap  CVFLDNFNISPMLFRELHQN--RTDAVGTARLNRKQIPNDLKK--RIAKGTTVARFCGELMALK-----W--CDGKEVTMLSTFHNDTVIEVNNRNGKKTKR--

pcPokeyS      SCPKAIDDYVNNMGGVDTANQLRSYYERDRKAKK-WWHRLLYSLLETCLVNSWICFNDMVEENYLENFEAPMTFLEFKRNVTMGLLSHALNEN---KNQAGRAG
pcPokeyL      SCPKAIDDYVNNMGGVDTANQLRSYYERDRKAKK-WWHRLLYSLLETCLVNSWICFNDIVEENYLEAYEVQMPFLEFKRNVTMGLLSHALNEN---KTKAGRAG
Pokey55       SCPKAIGDYVDNMGGVDTANQLRSYYERDRKSKK-WWHRLFYSLMETCMVNSWITYCDLVKGKTFLKDKRYLSLLEFKRSVTTSILFYWLNAE-KARKEDAPEA
Pokey3        SCPKAIGDYVDNMGGVDTANQLRSYYERDRKSKK-WWHRLFYSLMETCMVNSWITYCDLVKGKTFLKDKRYLSLLEFKRSVTTSLLLYGLNAE-KARKEDAPEA
Pokey21       SCPKAIDDYVNNMGGVDTANQLRSYYERDRKSKK-FWHRLLYSLLETCLVNSWVCFNDMVENNYLEYFEVQITFLEFKRNVTMGLLSNALNEN---KNKAGRAG
Pokey11       SCPKAIDDYVNNMGGVDTANQLRSYYERDRKAKK-WWHRLLYSLLETCLVNSWICFNDMVEENYLEHFEVPMTFLEFKRNVTMGLLSHALNET---KNQAGRAG
pB-Bmor       --PEMITFYNSTKAGVDVVDELSANYNVSRNSKR-WPMTLFYGVLNMAAINACIIYRA--------NKNVTIKRTEFIRSLGLSMIYEHLHS----RNKKKNIP
pB-Harm       --PDMILFYNQTKGGVDSFDQMCKSMSANRKTNR-WPMAVFYGMLNMAFVNSYIIYCH----NKINKQEKPISRKEFMKKLSIQLTTPWMQERLQAPTLKRTLR
pB-Xtro       --PLCSKEYSKYMGGVDRTDQLQHYYNATRKTRA-WYKKVGIYLIQMALRNSYIVYKAAVPGP-------KLSYYKYQLQILPALLFGGVEEQ----------
pB-like-Hsap  --PRVIVDYNENMGAVDSADQMLTSYPSERKRHKVWYKKFFHHLLHITVLNSYILFKK-------DNPEHTMSHINFRLALIERML----------EKHHKPGQ

                         NLS
pcPokeyS      RMMPTIHPSAEPGAKRRKSRLSVRDDIRFTGVGLHLPIFGEARGRCEWCQATT-----KTKLESRPFSSASNV---MCFCVSGRRE-IASSSIMMRITYLRRRL
pcPokeyL      RMMPTIHPSAEPGAKRRKSRLSVRDDIRLTCVGNHLPIFGEARGRCEWCQATT-----PKKLESRPFSKCKQC--NVFLCLGKKRN-C------FVEFHDDNYT
Pokey55       RLMKEIPLSAEPGAKRRKDRLSVPDEIRFSQVGIHHPIFVENRRRCEWCQATTERRPNGHTKESRPFSQCSMC--KIFLCLSKKRN-C------FLEFHDDRIL
Pokey3        RLMKEIPLSAEPGAKRRKDRLSVPDEIRFSQVGIHHPIFFGNRGRCEWCQATTERRPNGHTKESRPFSQCSMC--KIFLCLSKKRN-C------FLEFHDDRIL
Pokey21       RMMPTIHPSAEPGAKRRKSRLSVRDDIRFTGVGLHLPIFGEARGRCEWCQITT-----KTKLESRPFSKCKQC--NVFLCLGKKRN-C------FVEFHDENYL
Pokey11       RMMPTIHPSAEPGAKRRKSRLSVRDDIRFTGVGLHLPIFGEARGRCEWCQATT-----KTKLESRPFSKCKQC--NVFLCLGKRN-------------------
pB-Bmor       TYLRQ----------RIEKQLGEPSPRHVNVPGRYVR--------CQDC---------PYKKDRKTKHSCNAC--AKPICMEHAKFLC------ENCAELDSSL
pB-Harm       DNITNVLKNVVPASSENISNEPEPKKRRY----------------CGVC---------SYKKRRMTKAQCCKC--KKAICGEHNIDVC------QDCI------
pB-Xtro       -TVPEMPPSDNVA--RLIGK-HFIDTLPPTP-GKQRP----QKG-CKVCRKR------GIRRDTRYY--CPKCPRNPGLCFKP----C------FEIYHTQLHY
pB-like-Hsap  QHLRGRPCSDDVTPLRLSGR-HFPKSIPATS-GKQNP-----TGRCKICCSQYDKDGKKIRKETRYF--CAECC--DVPLCVVP----C------FEIYHTKKNY
```
