# Supplementary material for: In and out of the rRNA genes: characterization of Pokey elements in the sequenced Daphnia genome
Source: Mob DNA. 2013 Sep 23;4:20. doi: 10.1186/1759-8753-4-20 (PMC3849761; doi:10.1186/1759-8753-4-20)

Additional file 6. Repeated sequences in *Pokey* with similarity to *piggyBac* elements. The approximate location of repeat sequences in *piggyBac* that lack primary sequence identity with those in *Pokey*, but occur in similar locations are indicated for both elements. The dashed line in *Pokey* presents the repetitive region described in Figure 5. The repetitive region (dashed line), 5′ NCR and transposase genes (dashed boxes) are not drawn to scale. NCR = non-coding region, tpase = transposase gene.

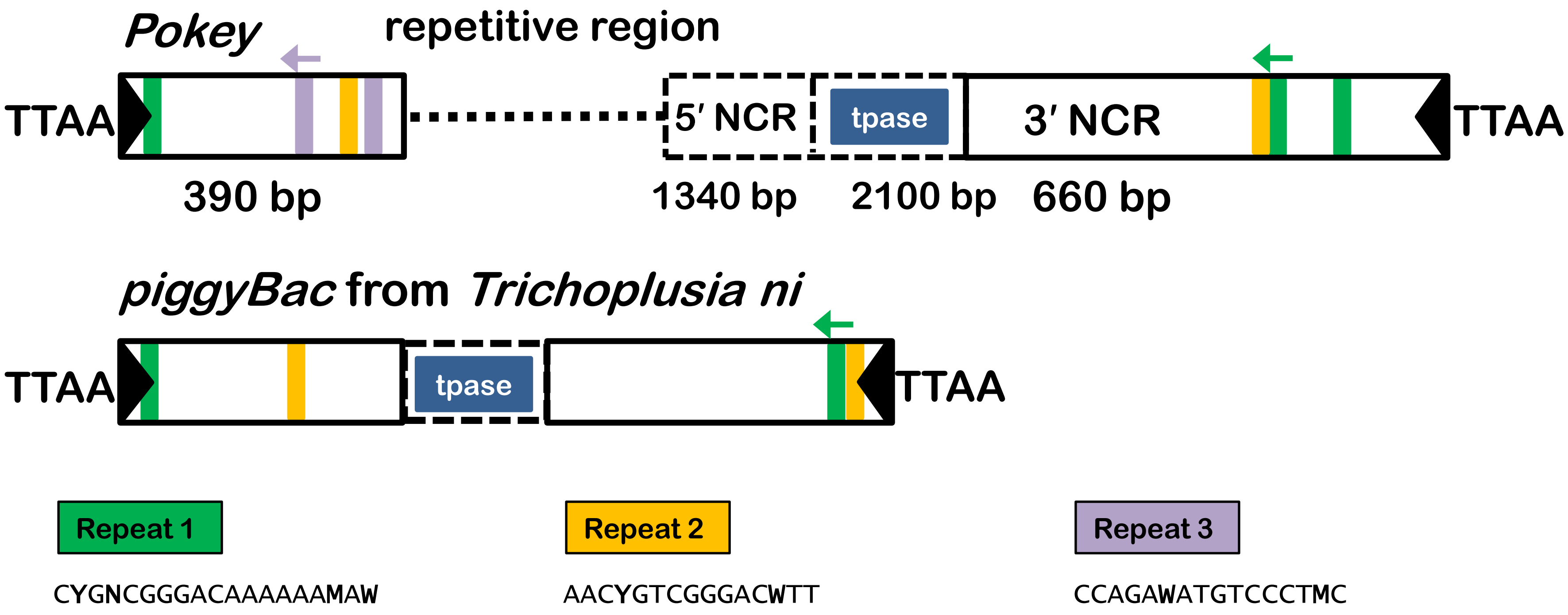

Supplement: Additional file 6 — Repeated sequences in Pokey with similarity to piggyBac elements. The approximate location of repeat sequences in piggyBac that lack primary sequence identity with those in Pokey, but occur in similar locations, are indicated for both elements. The dashed line in Pokey presents the repetitive region described in Figure 5. The repetitive region, 5′ NCR and transposase genes are not drawn to scale. NCR, non-coding region; tpase, transposase gene. [file 1759-8753-4-20-S6.pdf]
